# Resilience and burden in caregivers of older adults: moderating and mediating effects of perceived social support

**DOI:** 10.1186/s12888-018-1616-z

**Published:** 2018-01-31

**Authors:** Hui Lin Ong, Janhavi Ajit Vaingankar, Edimansyah Abdin, Rajeswari Sambasivam, Restria Fauziana, Min-En Tan, Siow Ann Chong, Richard Roshan Goveas, Peak Chiang Chiam, Mythily Subramaniam

**Affiliations:** 1 Department of Research Division, Institute of Mental Health, 10 Buangkok View, Hougang, 539747 Singapore; 2 Department of Geriatric Psychiatry, Institute of Mental Health, 10 Buangkok View, Hougang, 539747 Singapore

**Keywords:** Resilience, Social support, Caregiver burden, Older adults, Mental health, Singapore

## Abstract

**Background:**

The burden of caring for an older adult can be a form of stress and influence caregivers’ daily lives and health. Previous studies have reported that resilience and social support play an important role in reducing physical and psychological burden in caregivers. Thus, the present study aimed to examine whether perceived social support served as a possible protective factor of burden among caregivers of older adults in Singapore using moderation and mediation effects’ models.

**Methods:**

We conducted a cross-sectional study with 285 caregivers providing care to older adults aged 60 years and above who were diagnosed with physical and/or mental illness in Singapore. The Connor-Davidson Resilience Scale (CD-RISC) was used to measure resilience and burden was measured by the Zarit Burden Interview (ZBI). The Multidimensional Scale of Perceived Social Support (MSPSS) was used to measure perceived social support. Hayes’ PROCESS macro was used to test moderation and mediation effects of perceived social support in the relationship between resilience and burden after controlling for sociodemographic variables. Indirect effects were tested using bootstrapped confidence intervals (CI).

**Results:**

The mean scores observed were CD-RISC: 70.8/100 (SD = 15.1), MSPSS: 62.2/84 (SD = 12.2), and ZBI: 23.2/88 (SD = 16.0) respectively. While perceived social support served as a full mediator between resilience and caregiver burden (β = − 0.14, 95% CI -0.224 to − 0.072, *p* < 0.05), it did not show a significant moderating effect.

**Conclusions:**

Perceived social support mediates the association between resilience and caregiver burden among caregivers of older adults in Singapore. It is crucial for healthcare professionals, particularly those who interact and deliver services to assist caregivers, to promote and identify supportive family and friends’ network that may help to address caregiver burden.

## Background

In recent years there has been a growing interest in family caregiving of older adults. With technological advancements and vast improvements in healthcare services, the world’s population of older adults aged 60 years and above is expected to reach 2 billion by 2050 [1]. This rising figure implies an increasing burden and unmet need of informal caregivers of older adults [2]. Informal caregivers who are largely family members usually assist with basic and instrumental activities of daily living for older adults with common medical conditions associated with ageing such as dementia [3], and cancer [4].

The burden of caring for an older adult is well-documented in the existing literature [3, 5, 6]. The experience of providing care to them over a prolonged period can be a source of chronic stress and affect not only their caregivers’ daily lives and health [6–8], but also the society as a whole [8–10]. According to the Family Caregiver Alliance in the United States, the cost for businesses to replace female caregivers who quit their jobs due to caregiving duties has been estimated at $3.3 billion [9]. Furthermore, the estimated cost of replacing informal care with professional care services was reported to be at least $470 billion in 2013 [10]. In Singapore, the total annual societal cost of dementia was estimated to be SG$532 million [11]. Furthermore, it was projected that individuals with dementia would incur much higher societal cost than people without dementia [11]. Compared to the cost of formal caregiving, the mean annual cost of informal caregiving for people with dementia was much higher (SG$44,530.55 vs SG$25,654.11), largely due to the extra time spent by the informal caregivers [12]. Consequently, informal caregivers of older adults often experience a high level of care burden [5, 13], financial costs [14], and difficulties in coping with work and caregiving tasks [8, 13]. In Singapore, 8.8% of the informal caregivers of older adults had psychiatric morbidity, with a significant proportion having care-recipients with high care demand and severe behavioural and psychological symptoms of dementia [15]. With the considerable level of stress and burden involved in providing care, the concept of resilience and the availability of social support thus become crucial as they could function as protective factors to guard caregivers from the care burden.

### Resilience and social support

While some studies have documented poor physical and psychological health in caregivers [3, 15], there is evidence that caregivers also experience high satisfaction and positive returns from caregiving [16]. This disparity between observations across studies may be explained by other factors that could influence caregiver outcomes, such as resilience [17, 18] and social support [19, 20].

Resilience is defined as ‘positive adaptation to face adversity, flexibility, psychological well-being, strength, healthy life, burden, social network, and satisfaction with social support’ [17]. Two conceptual models of resilience posit that caregivers who have low resilience would experience high burden even in the presence of low care demand from a care-recipient. On the contrary, caregivers who have high resilience would experience low burden even when they experience high care demand [18]. This perceived low burden could be attributed to effective coping strategies where resilience was associated with problem- and emotion-focused coping strategies and sense of self-efficacy [21]. Therefore, resilience might be a key variable that explains the ability of some caregivers to ‘bounce back’ and deal with the challenges of caring for their loved ones [22].

Apart from resilience, various studies have supported the role of social support in protecting and maintaining physical and psychological health [20, 23]. For instance, social interaction and affective support help to reduce psychological burden in caregivers of patients with dementia [20]. While poor quality of social support can be detrimental and lead to adverse physical and mental health outcomes [24], positive experience of social support has been shown to foster resilience to stress and protect against psychopathology [25]. Other than informal relationships, social support could also be provided through caregiver’s formal relationships (e.g. family physicians, nurses, and social workers). However, a study found that informal social support, but not formal social support, was associated with lower caregiver burden [26]. There are two dimensions of social support namely; perceived social support and received social support. Perceived social support is viewed as ‘the perception of an individual about the amount and quality of support received from his/her social network’, while received social support is defined as ‘the objective quantification of the help and aid people receive from their social network’ [27, 28]. Research has shown that perceived social support is a stronger predictor of individual well-being than received social support and is closely related to personality traits such as optimism and self-esteem [29, 30].

Studies that have examined the relationship between resilience and social support, have not only found association between high resilience and hopefulness among cancer patients, but also shown positive influence of family social support on patients’ adaption process and longevity [31]. Having ‘open emotional expression and collaborative problem-solving’ is shown to improve family functioning under emotionally stressful situations experienced during caregiving which in turn improve informal social support, strengthen resilience and aid family adaptation [28].

To understand the associations between perceived social support, resilience, and caregiver burden, researchers have investigated inter-relationships that exist between them. Reduced perceived social support (as a measure of interpersonal resource) and low resilience (a measure of intrapersonal resource) were associated with acute post-traumatic stress disorder and dissociative symptoms [32]. Likewise, high perceived social support was associated with positive psychological well-being in times of stress [33].

To date, most research has addressed the dual relationships, either between resilience and burden, or social support and burden. Although a limited number of studies conducted in the United States of America and the United Kingdom have investigated the inter-relationships amongst the three factors [34, 35], these were largely conducted in Western populations. In Southeast Asia, a similar study in Malaysia found that resilience acts as a mediator between caregiver gender and burden [36]. Despite the vast research conducted on these factors across countries, little is known about the role of perceived social support as a moderator and/or mediator of the relationship between resilience (as a protective factor) and burden (as an outcome) in caregivers of older adults in Asian populations. It is possible that changes in burden may be directly influenced by the moderating effect of social support. As a result, people with high social support have higher resilience mechanisms which in-turn lead to lower caregiver burden and vice-versa. On the other hand, the association between resilience and caregiver burden may vary among those with low and high social support.

### Purpose and conceptual models

The purpose of this paper was to examine the relationship between resilience (a protective factor among caregivers) and caregiver burden as an outcome (a risk factor for poor physical and mental health). Furthermore, we aimed to investigate the role of perceived social support in moderating and/or mediating this relationship in informal caregivers.

We used conceptual models of moderation and mediation effects in this study [37]. In a moderation model, a moderator variable reduces or enhances the relationship between a predictor variable and an outcome variable, or it changes the direction of this relationship [37]. On the other hand, in mediation models, a mediator variable explains why a relationship exists between the predictor and outcome variables. In this study, we included social support as the moderating/mediating variable. The moderation model hypothesised that perceived social support would interact with resilience and influence the association between resilience and burden. The mediation model hypothesised that individual levels of perceived social support would facilitate the relationship between resilience and burden.

Given the rapid rate of ageing in Asian countries like Singapore [38] and the likely difference in the cultural values and practices of filial piety and familyism between Western and Asian societies [39], our study aimed to investigate the relationships between the three factors in a sample of Asian caregivers and address this research gap. Furthermore, if social support were found to be a substantial protective factor, it would be of utmost importance to develop appropriate interventions for caregivers with low social support. Guided by the conceptual models, we formulated two hypotheses– (1) perceived social support serves as a moderator; and (2) perceived social support serves as a mediator between resilience and burden among caregivers of older adults. The evidence of (1) and/or (2) would indicate that perceived social support functions as a protective factor of caregivers’ burden.

## Methods

### Participants

We conducted a cross-sectional study with self-reported data from informal caregivers of older adults across Singapore. Ethical approval for the study was given by the Domain Specific Review Board of the National Healthcare Group and written informed consent was obtained from all participants. To be eligible for the study, the caregiver had to be a Singapore citizen or permanent resident, aged 21 – 65 years, able to read and understand the questionnaire which was available in the English language, and currently providing care to an older adult aged 60 years and above. We excluded informal caregivers whose care-recipients were staying in nursing homes from the study. Two hundred eighty five informal caregivers who were providing care to an older adult with or without physical and/or mental illness completed the questionnaire. These participants were either referred by the psychiatrists treating care recipients at the Institute of Mental Health (IMH), or had participated in a previous study titled the Well-being of the Singapore (WiSE) study as an informant, and given their consent at that time to be re-contacted to participate in future research studies [40].

### Instruments

Sociodemographic information such as age, gender, ethnicity, marital status, educational level, employment status, and relationship of care-recipient to the caregiver was collected from participants.

#### Connor-Davidson Resilience Scale (CD-RISC)

The 25-item CD-RISC was administered to measure resilience [41]. All items on the CD-RISC used a five-point response scale (0 = ‘*not true at all’* to 4 = ‘*true nearly all the time’*). The scale is rated based on how the respondents had felt over the past one month and how much they agree with each item. Items include: ‘I can deal with whatever comes my way’. The total scores range from 0 to 100 with higher scores indicating higher resilience. Previous research has reported its high internal consistency (Cronbach’s alpha of 0.89), high test-retest reliability, and correlation with measures such as the Sheehan Social Support Scale (*r* = 0.36, *p* < 0.0001) [41]. CD-RISC had a Cronbach’s alpha of 0.94 in the current study.

#### Zarit Burden Interview (ZBI)

The 22-item ZBI was selected to measure caregiver burden in the study [42]. It is the fully revised version of the original 29-item scale [43] and has been used extensively in research conducted in caregivers of people with dementia [44, 45]. Participants rated on a five-point Likert response format (0 = ‘*never’* to 4 = ‘*nearly always’*). Items include: ‘Do you feel strained when you are around your relative?’ Total scores range from 0 to 88, with higher scores indicating a greater degree of burden. Studies have indicated that ZBI is a valid and reliable instrument to measure burden among caregivers of patients diagnosed with dementia and informal caregivers of community-dwelling older adults in Singapore [7, 46]. ZBI had a Cronbach’s alpha of 0.93 in the current study.

#### Multidimensional Scale of Perceived Social Support (MSPSS)

The 12-item MSPSS measured participants’ perceived social support from 3 informal sources: Family, Friends, and Significant Others [47]. Participants rated on a seven-point Likert response format (1 = ‘*very strongly disagree’* to 7 = ‘*very strongly agree’*). Items include: ‘I get the emotional help and support I need from my family’. The total scores range from 12 to 84, with higher scores indicating greater total perceived social support from all three sources. Zimet et al. [47] tested MSPSS and reported high internal consistency of 0.88. Test-retest reliability of 0.85 was reported over a 2 to 3 month period after completing the questionnaire [47]. MSPSS had a Cronbach’s alpha of 0.92 in the current study.

### Statistical approach

All statistical analyses were performed using the SPSS version 23.0. Two hundred seventy six cases with complete data on all three measures, CD-RISC, MSPSS and ZBI, were included in the analysis. We calculated the mean and standard errors for continuous variables and frequencies and percentages for categorical variables. We grouped age as a dichotomous variable for comparison between participants who were in the younger age group vs older age group (0 = ‘21-39’, 1 = ‘40-65’). Gender was dichotomised (0 = ‘Male’, 1 = ‘Female’). Ethnicity was coded as nominal variable (1 = ‘Chinese’, 2 = ‘Malay’, and 3 = ‘Indian’), as was marital status (1 = ‘Single’, 2 = ‘Married’, and 3 = ‘Separated/divorced/widowed’). Relationship of  care-recipient with the caregiver had four levels - parent, spouse, sibling and other. We converted ethnicity into three dummy coded variables: Chinese, Malay and Indian, with Chinese ethnicity treated as the reference group. Relationship was converted into four dummy coded variables: parent, spouse, sibling, and other, with the parent variable treated as the reference group.

Following Hayes [48] guidelines, We conducted SPSS PROCESS macro for testing hypotheses on the moderation and mediation effects. To test for the moderation effect (H_1_), the relationships for (i), (ii), and (iii) had to be significant - (i) direct effect of predictor (resilience) on burden, (ii) direct effect of moderator (social support) on burden, and (iii) direct interactions effect (resilience x social support) on burden. In SPSS PROCESS, interaction effect is calculated automatically via the software and it also produces the proportion of the variance explained by the moderating effect of perceived social support (R square increase due to interaction) [48].The mediating effect of perceived social support was tested in five steps (H_2_) - (i) direct effect of mediator (social support) on resilience, (ii) direct effect of predictor (resilience) on mediator (social support), (iii) total effect of predictor (resilience) on burden, (iv) direct effect of predictor (resilience) on burden with inclusion of mediator (social support), and (v) using SPSS PROCESS macro, a 1000-sample bootstrap procedure was used to estimate bias-corrected 95% confidence intervals (CIs) to test the significance of indirect effect of the relationships. If CIs do not contain 0, indirect relationships are significant, indicating significant mediating effect [48]. As mentioned by Hayes [49] and colleagues [50, 51], this bootstrapping procedure overcomes the limitations of the approaches highlighted by Baron et al. [37] and Sobel [52], yielding results that are more accurate and less affected by sample size [49–51]. Full mediation is presented when the beta weight is reduced and the *p*-value is not significant, while partial mediation is presented when the beta weight is reduced but the p-value is significant [37]. We adjusted for effect of the sociodemographic correlates (age, gender, ethnicity, and relationship to care-recipient) in the moderation and mediation models.

## Results

### Sample characteristics

Table 1 shows participants’ sociodemographic characteristics. The mean age of participants was 47 years, with the majority being  older (age group of 40-65) (73.7%), female (64.6%), Chinese (56.1%), married (60.7%), with tertiary level education (33.5%), and employed (75.8%). A majority of the care-recipients were a parent of the participants (78.6%), while the rest were other relatives/other (14.4%), spouse (6.3%), or sibling (0.7%). Majority of the care-recipients were either diagnosed as having physical illnesses such as hypertension, hyperlipidaemia, diabetes mellitus, and/or mental illnesses including dementia.

**Table 1:** Sociodemographic profile of the participants (*N* = 285)

		*n*	%
Age group	21-39	75	26.3
	40-65	210	73.7
Gender	Male	101	35.4
	Female	184	64.6
Ethnicity	Chinese	160	56.1
	Malay	38	13.3
	Indian	87	30.5
Marital status	Single	85	29.8
	Married	173	60.7
	Separated/Divorced/Widowed	27	9.5
Education level	Completed Primary	9	3.2
	Completed Secondary	76	26.8
	Completed Vocational Education	13	4.6
	Completed A Level	17	6.0
	Completed Diploma	74	26.1
	Completed Tertiary Education	95	33.5
Employment status	Employed	216	75.8
	Unemployed	69	24.2
Relationship of care-recipient with caregiver	Parent	224	78.6
	Spouse	18	6.3
	Sibling	2	0.7
	Other relatives/ others	41	14.4

#### Conceptual variables

Table 2 shows the correlations, means, and standard deviations of the three measures in the current sample. The mean score of CD-RISC was 70.8 (SD = 15.05). Mean scores of ZBI and MSPSS were 23.2 (SD = 15.98) and 62.2 (SD = 12.23), respectively.

**Table 2:** Correlations, means, standard deviations of the three measures

			Correlation Matrix		
Measures	Mean	SD	1	2	3
1. Burden	23.2	15.98	─		
2. Resilience	70.8	15.05	-0.15^a^	─	
3. Perceived social support	62.2	12.23	-0.31^a^	0.40^a^	─

^a^Correlation is significant at the 0.01 level (2-tailed)

#### Moderation and mediation effect of perceived social support

**Fig. 1:**
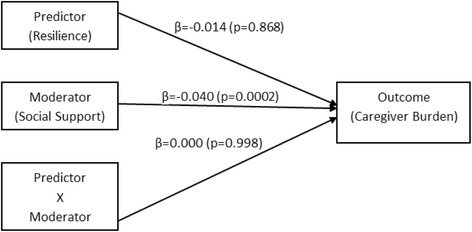
Output Model of Perceived Social Support Moderation

A series of analyses were conducted to test the first hypothesis on moderating effect of perceived social support (Table 3). Perceived social support did not demonstrate moderating effect as there was no significant association between resilience and burden (β = − 0.014, *p* > 0.05), and no interaction effect of resilience and perceived social support on burden (β = 0.000, *p* > 0.05). Figure 1 illustrates the output model for the moderation effect of perceived social support.

**Table 3:** Results from PROCESS macro testing perceived social support moderation model

Effect^a^, Variable	R^2^	*F*	β	*p*
i. Direct effect of predictor (resilience) on burden	0.111	− 0.167	− 0.014	0.868
ii. Direct effect of moderator (perceived social support) on burden	0.111	−3.785	−0.402	< 0.001
iii. Direct interaction effect (resilience x perceived social support) on burden	0.111	−0.003	0.000	0.998

^a^Adjusted for age, gender, ethnicity, and caregiver-care recipient relationship

**Fig. 2:**
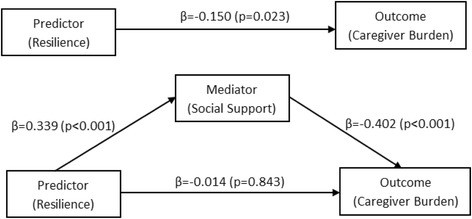
Output Model of Perceived Social Support Mediation

Perceived social support demonstrated mediating effect whereby the indirect effect of resilience on burden through perceived social support (mediator) was statistically significant (β = − 0.136, 95% CI -0.224 to − 0.072) (Table 4). Figure 2 illustrates the output model for the mediation effect of perceived social support.

**Table 4:** Results from PROCESS macro testing perceived social support mediation model

Effect^a^, Variable	R^2^	*F*	β	*p*
i. Direct effect of mediator (perceived social support) on burden	0.111	−4.716	−0.402	< 0.001
ii. Direct effect of predictor (resilience) on mediator (perceived social support)	0.208	7.484	0.339	< 0.001
iii. Total effect of predictor (resilience) on burden	0.037	−2.289	−0.150	0.023
iv. Direct effect of predictor (resilience) on burden with inclusion of the mediator (perceived social support)	0.111	−0.199	−0.014	0.843
	β	95% CI		*p*
v. Indirect effect of predictor (resilience) on burden	−0.136	− 0.224	−0.072	< 0.05

^a^Adjusted for age, gender, ethnicity, and caregiver-care recipient relationship

## Discussion

The focus of this paper was perceived social support and its ability to moderate and/or mediate the relationship between resilience and caregiver burden among informal caregivers of older adults. Our results describe for the first time the relationships between these three important experiences in Singapore’s caregiver population. We found that the significant association between resilience and caregiver burden was mediated by the level of perceived social support. However, perceived social support did not moderate this relationship.

These results are in agreement with other studies that have shown that caregivers with higher level of social support experience lower level of burden [19, 43], which could be attributed to the ‘buffering model’ whereby social support acts as a buffer against stress by attenuating or preventing the stress appraisal response of caregivers [24].

Our findings indicate that resilience and perceived social support contribute significantly to caregivers’ burden. Findings from the mediation model (Fig. 2) showed that perceived social support acts as a mediator or third variable, whereby upon addition of perceived social support into the model, reduces the beta weight of resilience rendering it ineffective/non-significant in predicting the caregiver burden. This reduction in beta weight and insignificance reflects the full mediating effect of perceived social support on the relationship between resilience and burden [37].

In contrast to some reports in the literature [17, 53], we did not find moderation effect in this study. Based on the theory of moderation proposed by Frazier et al. [54], this indicates that perceived social support does not influence the direction of the relationship between resilience and caregiver burden.

### Research implications

Our findings bear significant implications for healthcare practitioners and professionals who work with caregivers of older adults. The full mediating effect of perceived social support offers clear implications for practice and policy. Consistent with other studies, this study highlights the importance of perceived social support [19, 55]. The significant inverse relationship that has emerged between caregiver burden and perceived social support suggests that caregiver burden is dependent on their level of perceived social support. For a caregiver, having a family member or friend to discuss their problems with is beneficial. In a qualitative study conducted among caregivers who were caring for their family members or relatives, 62% identified lack of social support as one of their challenges to caregiving. Moreover, 83% identified emotional support (i.e. seeking out to friends and family or others) to be effective in dealing with these challenges [14]. Family caregivers who are resilient were more likely to adopt positive coping skills, express their concerns and find sympathetic listeners [56]. The act of venting their concerns and emotions can have a calming effect that is essential for caregivers’ mental and emotional well-being [57].

In addition to enhancing social support at an individual level, community support services such as home help, mobile medical services, and long-term care services should be available to family caregivers. In Singapore, there are several caregiver support programmes, particularly for caregivers of older adults, such as Family of Wisdom Programme (FOW) by Alzheimer’s disease Association (ADA), Asian Women’s Welfare Association (AWWA), and TOUCH caregivers support (TCG). As reported by Lopez-Hartmann et al. [58], group support has a positive impact on caregivers’ coping ability, knowledge and social support and helps in reducing depression. Thus, community support programmes in Singapore could be expanded to assist the caregivers.

Previous studies have reported some effective interventions for enhancing resilience among individuals [17, 41]. Resilience training which cultivates problem-solving skills and conflicts’ resolution was found to be helpful in equipping caregivers with skills to cope and reduce depression [59]. Medication such as receiving an antidepressant drug, escitalopram, and interventions like cognitive behavioural therapy (CBT), and mindfulness were suggested to be effective in building resilience in individuals and caregivers of relatives with Alzheimer’s disease [60, 61]. By enhancing happiness and optimism, positive thinking helps to strengthen one’s resilience and well-being more efficiently [22].

The present study however has some limitations. Firstly, only Singapore residents were invited to take part in the study, and thus the generalizability of the results to other populations is limited. Secondly, study inclusion was limited to caregivers who were able to read and understand the English questionnaire in the study. The lack of other language questionnaires thus has limited generalizability for caregivers with low education or those educated in their mother-tongue languages such as Chinese, Malay or Tamil. Thirdly, the study did not evaluate other protective factors such as positive emotions, private prayer, and physical health, which have also been shown to influence the relationship between resilience and caregiver burden [22, 34, 62]. Finally, social desirability bias among participants should be considered. In this study conducted in an Asian setting, caregivers who were the children of care-recipient may have answered questions on ZBI more positively as they may have perceived it being viewed favourably by others as being filial. In Asian societies, providing care and financial support to one’s parents is considered one of the critical components of filial piety [63]. To reduce this bias, interviewers reassured all participants that there were no right or wrong answers and were instructed to answer all questions based on their first instinct to minimise the effect of elucidating socially desirable responses. Moreover, we adjusted for age, gender, ethnicity, and relationship with care-recipients in the moderation and mediation analyses.

Notwithstanding these limitations, our study draws attention to the significance of the effect of perceived social support on the association between resilience and caregiver burden relationship in caregivers of older adults in Singapore. Future research will be needed to look at other potential confounders. For instance, the length of caregiving and adequate sample diversity such as the inclusion of different types of caregivers, e.g. sole caregivers, full-time caregivers, caregivers with low financial incomes or education level, and spousal/non-spousal caregivers. Also, it would be desirable to replicate this study with longitudinal data and to examine the strength of perceived social support as a moderator/mediator across caregivers of care-recipients with different types of physical and/or mental illnesses.

## Conclusions

In this paper, we explored the possibility that perceived social support functioned as a protective factor of care burden. Results showed that perceived social support mediates the relationship between resilience and caregiver burden. The findings of our research are of direct practical relevance and provide valuable insight into the relationship between perceived social support, resilience, and burden among the caregivers of older adults in Singapore. Perceiving social support from family, friends, and significant others is beneficial for caregivers, and thus should be recognised and promoted by healthcare professionals who have regular contact with the caregivers and family members of older adults.

## Abbreviations

CD-RISC: Connor-Davidson Resilience Scale
MSPSS: Multidimensional Scale of Perceived Social Support
ZBI: Zarit Caregiver Burden Interview
